# Plio-Pleistocene evolution of Bohai Basin (East Asia): demise of Bohai Paleolake and transition to marine environment

**DOI:** 10.1038/srep29403

**Published:** 2016-07-07

**Authors:** Liang Yi, Chenglong Deng, Lizhu Tian, Xingyong Xu, Xingyu Jiang, Xiaoke Qiang, Huafeng Qin, Junyi Ge, Guangquan Chen, Qiao Su, Yanping Chen, Xuefa Shi, Qiang Xie, Hongjun Yu, Rixiang Zhu

**Affiliations:** 1 State Key Laboratory of Marine Geology, Tongji University, Shanghai 200092, China; 2 Institute of Deep-sea Science and Engineering, Chinese Academy of Sciences, Sanya 572000, China; 3 Key Laboratory of Marine Sedimentology and Environmental Geology, First Institute of Oceanography, State Oceanic Administration, Qingdao 266061, China; 4 State Key Laboratory of Lithospheric Evolution, Institute of Geology and Geophysics, Chinese Academy of Sciences, Beijing 100029, China; 5 University of Chinese Academy of Sciences, Beijing 100049, China; 6 Key Laboratory of Muddy Coastal Geo-Environment, Tianjin Centre, China Geological Survey, Tianjin 300170, China; 7 State Key Laboratory of Loess and Quaternary Geology, Institute of Earth Environment, Chinese Academy of Sciences, Xi’an 710061, China; 8 Key Laboratory of Vertebrate Evolution and Human Origins of the Chinese Academy of Sciences, Institute of Vertebrate Paleontology and Paleoanthropology, Chinese Academy of Sciences, Beijing 100044, China; 9 Key Laboratory of Engineering Oceanography, Second Institute of Oceanography, State Oceanic Administration, Hangzhou 310012, China; 10 National Deep Sea Center of China, Qingdao 266061, China

## Abstract

The Bohai Basin was transformed to an inner shelf sea hundreds of thousands years ago. This youngest land-sea transition participated in the significant modification of the distribution of fresh water, sediment fluxes and climate in East Asia, and played an important role in the origin of the Asian marginal seas. Here we present the results of a magnetostratigraphic investigation and propose a conceptual model for the land-sea transition. Our findings indicate that the transition probably started several million years ago, from a fluvial system during the late Miocene and early Pliocene, to a lacustrine environment between the late Pliocene and Middle Pleistocene, and finally to a marine system in the late Pleistocene. Comparison of our results with previous research suggests that the Bohai Paleolake was initiated from the late Pliocene, was fully developed prior to ~1.0 Ma, and terminated around the late Middle Pleistocene. The Miaodao Islands formed the eastern “barrier” of the basin and since the Pliocene or earlier they played a significant role in blocking the lake water and sediments. They deformed from ~1.0 Ma, subsided significantly at ~0.3 Ma and completely by ~0.1 Ma, resulting in the maturation of the basin as an inner shelf sea.

As a result of the Cenozoic deformation of the Asian continent and subduction of the Pacific plate, a series of marginal seas, from the Bering Sea in the north to the Banda Sea in the south, were separated from the Asian continent^1^. The presence of these marginal seas caused significant modification of the material and energy flux linkages between Asia and Northwest Pacific, and which has attracted considerable research interest over the past decades^1^,^2^,^3^,^4^. The Bohai Sea and Yellow Sea are affiliated to the East China Sea, and as the youngest part, they provide a critical window for observing the pre-opening history and sea-land interaction of the marginal seas, while relevant sedimentary records in the other parts were too deeply buried to be accessible^5^,^6^.

Prior to the existence of a continental shelf environment, the region of the Bohai Sea and Yellow Sea was isolated as several sedimentary basins^6^. The basins were constrained by three major paleo-uplifts, similar to a three-order dam system. This system contained four major basins (Fig. 1): the North Jiangsu–South Yellow–Sea basin, Qing–Dong basin, North Yellow–Sea basin, and Bohai basin.

The Bohai basin is the third-order reservoir constrained by the Miaodao Uplift, and was formed by subsidence during the Cenozoic^5^,^7^,^8^. Approximately 2000–3000 m of fluvial, lacustrine, and marine sediments have been deposited in the basin^9^. Since the 1970s, hundreds of cores have been drilled around the Bohai Sea for the purpose of geological, hydrological, and natural resources research, and a major finding is evidence for alternations between transgression and regression during the Pleistocene. A classical study based on 71 coastal cores^10^ proposed that there were three transgressions in the western Bohai Sea, which can be correlated to the late Pleistocene (since 130 ka). IOCAS ref. 9 further divided the three major transgressions into seven sea-level events based on Borehole BC-1 from the Bozhong basin (Fig. 1). Since the 1990s, considerable efforts have been made to understand the environmental and geological evolution of the basin, and the number of long boreholes drilled around the Bohai Sea has increased dramatically^11^,^12^,^13^,^14^,^15^,^16^. Recently, Yi *et al*. ref. 17 re-dated the three major transgression events proposed by Zhao *et al*. ref. 10 and argued that these events occurred significantly earlier than proposed in previous studies. However, since very few geochronological frameworks have been established for the pre-Bohai Sea interval, the Bohai Paleolake (BHPL) prior to 260 ka^15^ was discussed mainly from a sedimentological perspective^6^,^9^,^11^,^18^,^19^,^20^, and there are no reports examining the timing of the formation of the BHPL and its long-term evolution.

The present study uses magnetostratigraphy to date three sedimentary sequences (Boreholes BH1, BH2 and HLL02) from the southern Bohai Sea, spanning the Pliocene to the Pleistocene Epochs (Figs 2 and 3). A chronostratigraphic framework is established for the Bohai basin since the Pliocene by combining the sedimentary characteristics and geochronological information from three new boreholes and integrating the findings with those of previous works. A conceptual model is subsequently proposed to reveal the possible timing of initiation and processes of evolution of the BHPL (Figs 4 and 5). Since there were several periods of lake development during the Meso-Cenozoic^9^,^20^, this study concentrates on the final period of lake development, spanning the interval from its probable origin in the late Miocene to its transformation to an inner continental shelf environment in the late Quaternary.

## Results

Stepwise demagnetization was conducted on 1756 samples of the three borehole sequences. In general a secondary magnetization component, probably of viscous origin, was present and was removed by thermal demagnetization (TD) below 150 °C or by alternating field demagnetization (AFD) below 20 mT (Supplementary Fig. S4). For most samples, a high-stability characteristic remanent magnetization (ChRM) component was separated between 20 mT and 50 mT or between 300 °C and 585 °C with maximum angular deviation (MAD) values of ≤15°. In a few of the samples, the ChRM component was separated between 610 °C and 690 °C also with MAD values of ≤15°. A total of 1199 samples (68%) provided reliable ChRM directions (Supplementary Table S1). There was no statistically significant difference between the results of thermal or alternating field demagnetization and the paleo-latitudes were close to the modern geographical latitude (Supplementary Fig. S5 and Table S2). A minimum of four consistently normally or reversely magnetized samples were used to define magnetozones. The criteria resulted in the recognition of eight, seven, and seventeen magnetozones for Boreholes BH1, BH2, and HLL02, respectively (Fig. 2; Supplementary Fig. S5).

The polarity sequences can be correlated to the Astronomically Tuned Neogene Time Scale (ATNTS2012)^21^ by combining magnetostratigraphic and sedimentary evidence. The correlation suggests that the sediments record geomagnetic polarity sequences ranging from the Gilbert reverse chron to the Brunhes normal chron (Fig. 2). The results from individual cores are summarized as follows:

1. *Borehole BH1*. Four normal magnetozones N1, N2, N3, and N4, are recorded which correspond to the Brunhes chron (C1n), Jaramillo subchron (C1r.1n), Olduvai chron (C2n), and late Gauss chron (C2An.1n), respectively. Then corresponding reverse magnetozones R1–R3 can be correlated to the successive reversed polarity intervals of the intervening Matuyama chron, while magnetozone R4 can be correlated to the Kaena subchron (C2An.1r).
2. *Borehole BH2*. Four normal magnetozones (N1–N4) can be correlated to the Brunhes chron (C1n), Jaramillo subchron (C1r.1n), Olduvai chron (C2n), and late Gauss chron (C2An.1n), respectively. Reverse magnetozones R1–R3 can be correlated to the intervening Matuyama chron.
3. *Borehole HLL02*. Eight normal magnetozones (N1, N3–N9) can be correlated to the Brunhes chron (C1n), Olduvai chron (C2n), Gauss chron (C2An), Cochiti subchron (C3n.1n), Nunivak subchron (C3n.2n), Sidufjall subchron (C3n.3n), and Thvera subchron (C3n.4n), respectively. Reverse magnetozones R1–R3 and R5–R8 can be correlated to the Matuyama and Gilbert reverse chrons, respectively.

## Discussion

The integrated magnetostratigraphic results and previously-published luminescence ages^17^ enable the establishment of a chronostratigraphical framework for the Plio-Pleistocene marine and terrestrial strata in the study area (Supplementary Table S3).

The average sediment accumulation rates (SARs) exhibit three distinct stages corresponding to significant changes in depositional environment (Fig. 3; Supplementary Fig. S6): (1) Phase I (prior to 3.7 Ma) had an average SAR of 185 m/Myr and the sedimentary environment was fluvial; (2) Phase II (3.7–0.26 Ma) had an average SAR of 40–60 m/Myr and the sedimentary environment was lacustrine; (3) Phase III (0.26–0.01 Ma) had an average SAR of 140–180 m/Myr and the sedimentary environment was marine. Extrapolation of the ages of the fluvial and lacustrine phases results in an ages of ~3.7 Ma for the intersection between the two, which is consistent with the age estimates of the sedimentary transition at 196 m (Supplementary Fig. S7). This suggests that the extrapolated age of ~3.7 Ma for the origin of lake is reliable.

The reported ages of the transition from a lacustrine to a marine environment vary considerably: from marine isotope stage (MIS) 5 (130–71 ka) base on extrapolation of radiocarbon dates^2^,^9^,^11^ to MIS 7 (243–191 ka) or older based on luminescence or uranium-series dating^16^,^17^,^22^,^23^ and magnetostratigraphy^13^,^24^. To date, no absolute age constraints have been provided; however, Yi *et al*. ref. 15 compared ages obtained using different independent methods and concluded that the transition probably occurred at around 0.3 Ma. Backward extrapolation of the OSL-based age-depth model for Borehole BH1 and BH2^17^ provided an age of ~0.3 Ma for this transition (Supplementary Table S4). This was also close to the age produced by the forward extrapolation of the paleomagnetic ages reported in this study, probably confirming that the lake-to-sea transition occurred at ~0.3 Ma (Supplementary Fig. S7).

The Bohai basin experienced several intervals of lake development during the Meso-Cenozoic^9^, while the sedimentary environment was mainly fluvial^20^ during the intervening intervals. Based on the reliable geochronological information and changes in sedimentary facies, we propose the following conceptual model for depicting the evolutionary process of the Bohai basin since the early Pliocene.

Prior to ~3.7 Ma, fluvial deposition dominated around Laizhou Bay (Fig. 4). Coarse-grained sediments and a high SAR suggest rapid subsidence in the basin and/or uplift of the surrounding mountains as the dominant controls on sedimentation. According to Borehole HLL02, this fluvially dominated interval commenced either around 5.1 Ma (Fig. 3) or around 6.5 Ma based on evidence from a 452-m-long core located several tens of kilometers away from Borehole HLL02 (Jiang XY, unpublished data). A 1226-m-long core from Bohai Bay dated to 8.1 Ma by magnetostratigraphy^25^ contains coarse-to-medium sand with gravel clasts commonly distributed throughout the middle and lower parts. In addition, a relatively high SAR interval prior to 3.0 Ma is reported^25^. Consequently, we infer that during the late Miocene and the early Pliocene the Bohai basin experienced rapid subsidence relative to the surrounding mountains. This caused an increase in fluvial gradient and an intensification of sedimentary dynamics which resulted in the development of the fluvial system around the basin.

Compared with that of the interval dominated by fluvial sedimentation, the SAR in the subsequent lacustrine phase was significantly reduced, from 162 m/Myr to 40–60 m/Myr (Fig. 3). Although the initial timing was different in each sub-basin (Fig. 4), lacustrine deposition developed around the Bohai basin mainly from the late Pliocene. This strongly suggests the gradual subsidence of the Bohai basin and continuous lake development, namely the initiation of the BHPL. The lacustrine phase can be roughly divided into three sub-stages according to sedimentary changes (Fig. 4): (1) In the sub-stage prior to the Olduvai chron (C2n), the regional sedimentary environment alternated between longer intervals of lake development and shorter intervals in which a fluvial system predominated; (2) prior to the Jaramillo subchron (C1r.1n), the lake developed continuously without major interruptions or marine transgressions; and (3) prior to ~0.3 Ma, several “weak” transgressions occurred which were characterized by the low diversity and abundance of foraminifera^26^. The sub-stages are evident not only in Laizhou Bay but also in Bohai Bay and the Bozhong basin (Fig. 4). For example, a continuous sequence of lake sediments was deposited in western Bohai Bay from ~2.2 Ma^13^, and the sedimentary environment in northern Bohai Bay alternated between lake- and fluvially-dominated prior to the Olduvai chron (C2n), but became dominantly a lake system thereafter^27^. In the Bozhong basin, results from several long boreholes have been reported; however, the only two recent well-dated long cores include Boreholes BH08^14^ and TCJ1^28^. As illustrated in Fig. 4, although the SAR exhibits significant variations between cores, both cores from the Bozhong basin record a continuous lake environment in their middle and lower sections. Thus the results from all three major parts of the Bohai basin are comparable and suggest three stages in the evolution of the BHPL.

Since ~0.3 Ma, the BHPL terminated due to the subsidence of the Miaodao Uplift^15^, resulting in the development of the Bohai basin as an inner shelf sea. In addition, the three major transgressions proposed in the literature^10^ occurred in the context of global sea-level changes.

Sea-level changes are expected to have influenced the evolution of the Bohai basin^15^. However, it can also be inferred that regional tectonic activity, including the subsidence of the Bohai basin and/or the uplift of its surrounding mountains, and the Miaodao Uplift (Islands) that constituted the major barrier between the Bohai and the North Yellow-Sea basins, played first-order roles in this process. Based on the foregoing discussion, it is possible tentatively to infer the roles played by the Miaodao Uplift during the different stages of the evolution of the BHPL (Fig. 5).

Since the late Miocene, or even earlier, a fluvial system dominated the Bohai basin. Major regional rivers, such as the Liaohe, Haihe, Luanhe, Huanghe, Xiaoqinghe, and Mihe Rivers, discharged into the basin; however, no persistent mega-lake was developed, which may indicate that the Bohai and the Yellow-Sea basins were inter-connected and thus that a large volume of fresh water could not be stored in the Bohai basin (Fig. 5a).

Since the late Pliocene, the Bohai basin was isolated due to the continuous subsidence of the basin and/or the uplift of the surrounding mountains (including the Miaodao Uplift). Freshwater transported by the regional rivers could not drain from the basin, and the accumulated lake water reached a higher level compared to the present level (Fig. 5b,c). However, the elevation of the Miaodao Uplift gradually diminished, with the increase in the volume of lake water and accumulated sediments within the basin, together with weakened tectonic activity. Recent reports of transgression events around the Bohai basin^14^,^28^ suggest that the well-dated oldest transgression occurred within the Jaramillo subchron (C1r.1n), or slightly later, and that these transgressions were all “weak”. Furthermore, the local maxima in the lake water level were approximately 0.5 m or higher prior to the Jaramillo subchron (C1r.1n), compared to the subsequent local lake water maximum levels^15^. It is speculated that the Bohai and the Yellow-Sea basins were inter-connected again at ~1.0 Ma by several channels (Fig. 5d).

Since the late Middle Pleistocene, three major transgressions around the Bohai basin occurred. The water level fluctuated frequently but with an overall continuously decreasing trend^15^, indicating that the “barrier” Miaodao Uplift had significantly subsided (Fig. 5e). The Miaodao Uplift subsided completely after ~0.1 Ma. The regional water level varied with global changes^15^, and the Bohai basin developed subsequently as an inner shelf sea (Fig. 5f).

In summary, the BHPL probably originated in the late Pliocene, and its subsequent evolution occurred in three stages. As the eastern “barrier” of the basin, the Miaodao Uplift played a significant role since the Pliocene or earlier. During the BHPL development, from the late Pliocene, it dammed the lake water and sediments, before diminishing at ~1.0 Ma. It significantly subsided after ~0.3 Ma and completely subsided at ~0.1 Ma, resulting in the maturation of the Bohai basin as an inner shelf sea. It is clear that the study only presents an outline of the land-sea transition of the Bohai basin from a geochronological perspective, without considering possible differences between the sub-basins. Hence, further investigations are required to validate the proposed model.

## Methods

**Figure 1:**
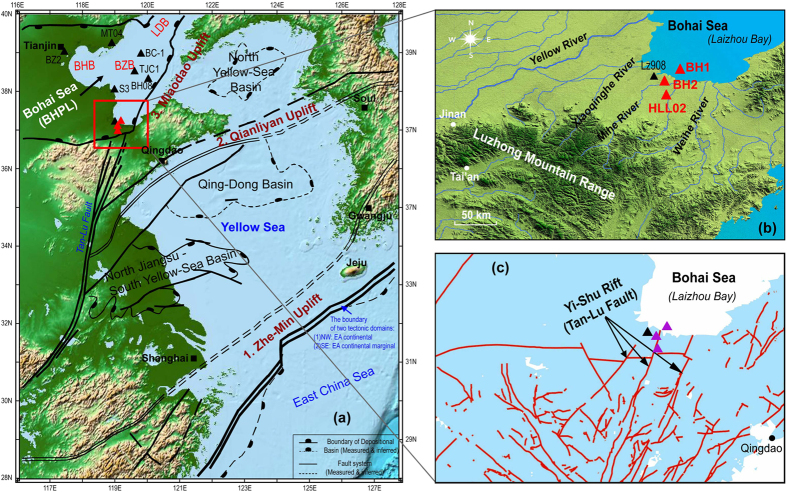
Map showing the location of Boreholes BH1, BH2 and HLL02 (red triangles) in the Bohai Sea. BHB, Bohai Bay; LDB, Liaodong Bay; BZB, Bozhong basin. The basins and faults are redrawn from ref. 34. Some boreholes mentioned in the text are labeled black triangles. The base map data (**a,b**) was generated using the open and free software DIVA-GIS 7.5 (http://www.diva-gis.org/).

**Figure 2:**
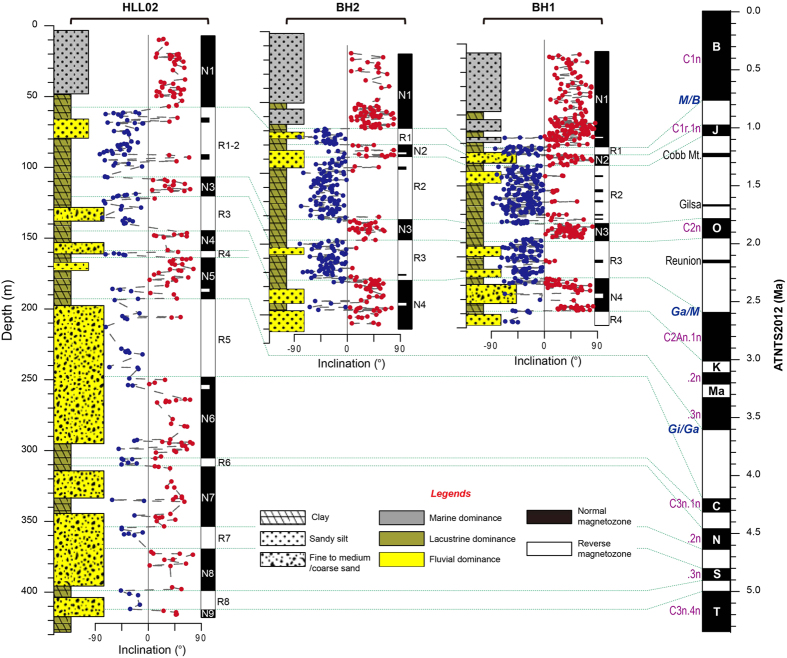
Lithostratigraphy and magnetostratigraphy of Boreholes BH1, BH2, and HLL02 from the southern Bohai Sea. ChRMs obtained using different demagnetization methods are labeled in Supplementary Fig. S5, and the results of statistical tests are listed in Supplementary Table S2. The dotted lines (right panel) indicate possible correlations of the recognized magnetozones to the ATNTS2012 ref. 21. B, Brunhes; M, Matuyama; O, Olduvai; Ga, Gauss; K, Kaena; Ma, Mammoth; Gi, Gilbert; C, Cochiti; N, Nunivak; S, Sidufjall; T, Thvera.

**Figure 3:**
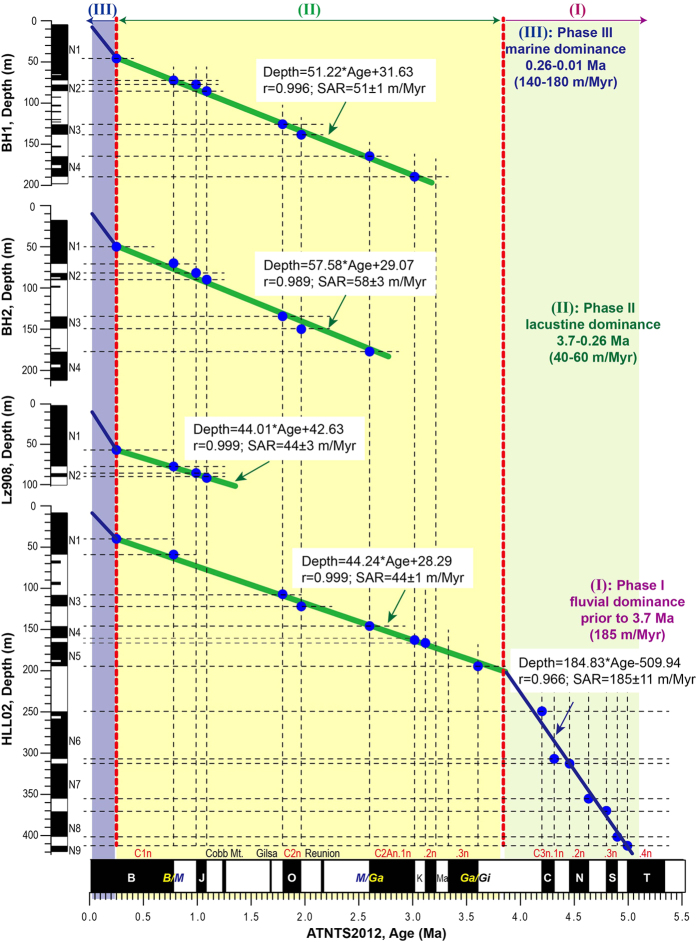
Geochronology and sediment accumulation rate (SAR) variations recorded in Boreholes BH1, BH2 and HLL02 since the early Pliocene. Three phases (I, II and III) with their average SARs and age-depth models are displayed. The magnetostratigraphy of Borehole Lz908 ref. 15 and ATNTS2012 ref. 21 are shown for reference. Also see Supplementary Figs S6–S7 and Tables S2–S3.

**Figure 4:**
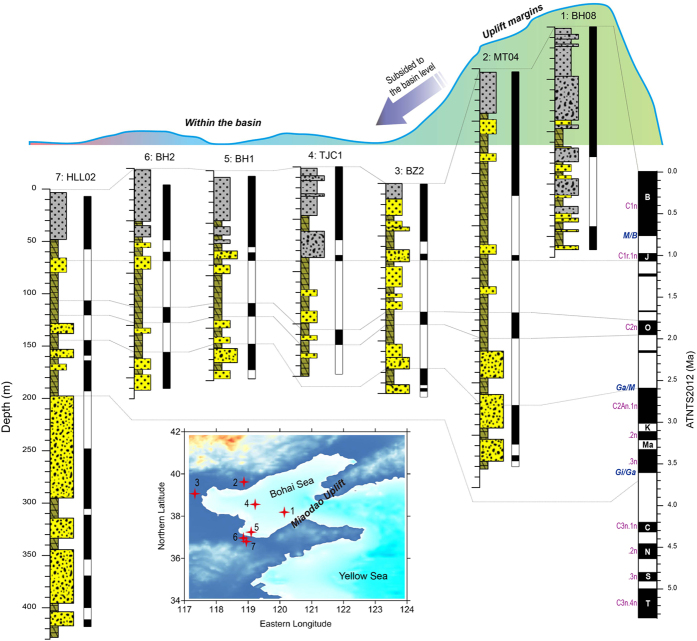
Lithological and magnetostratigraphic correlations around the Bohai Sea. Boreholes BH1, BH2, and HLL02 are from this study, and MT04 ref. 27, BZ2 ref. 13, BH08 ref. 14, and TCJ1 ref. 28 are cited from previous studies. The base map data showing the core locations was generated using the open and free software DIVA-GIS 7.5 (http://www.diva-gis.org/).

**Figure 5:**
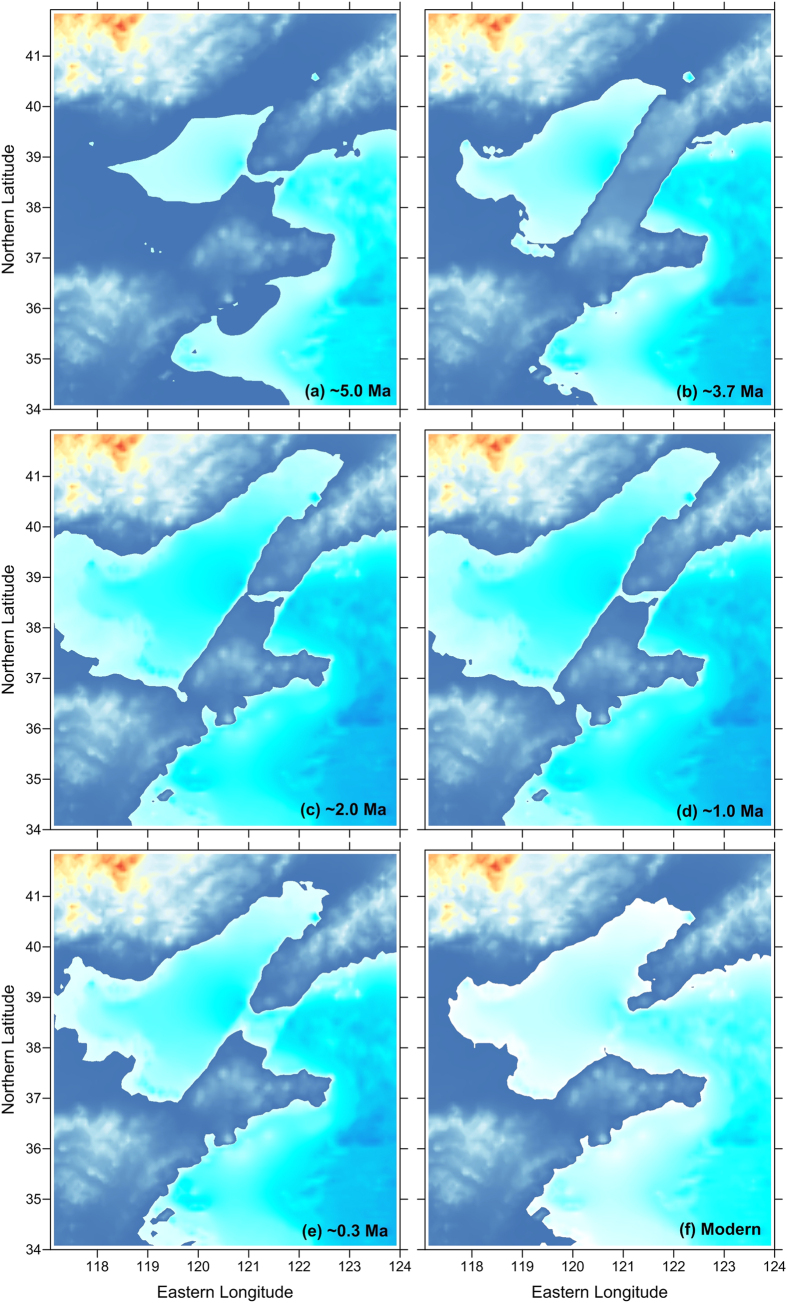
A conceptual model for geomorphological evolution of the Bohai basin and Miaodao Islands (Uplift) since 5.0 Ma. Spatial differences in tectonic subsidence and depositional processes are not considered. The base map data was generated and reanalyzed using the open and free software DIVA-GIS 7.5 (http://www.diva-gis.org/).

The southern Bohai Sea is located in the Yi-Shu Rift^29^,^30^, and the interval from the Neogene to the present was characterized by persistent subsidence and continuous sediment accumulation^31^,^32^. The sediments deposited in the southern Bohai Sea were mainly transported from the Luzhong Mountain Range by local rivers^16^, such as the Mihe River, Xiaoqinghe River, and Weihe River (Fig. 1). These rivers are only 100–300 km in length, and thus the depositional processes can be expected to have been relatively uniform and continuous, given the close proximity of the sediment source and sustained tectonic subsidence.

Boreholes BH1 (37°17′N, 119°06′E, water depth −4 m, length 199 m), BH2 (37°10′N, 119°04′E, elevation 3 m a.s.l., length 228 m), and HLL02 (37°02′N, 119°08′E, elevation 3 m a.s.l., length 425 m) are employed in this study. On the basis of sedimentary characteristics (Supplementary Figs S1–S3), three depositional units (DU) are identified, designated DU–1, DU–2 and DU–3 in ascending order.

1. DU–1 contains massive yellowish, olive-gray, brown-gray and light-gray fine to medium or coarse sand and thin gray, brown-gray and olive-gray clay. The coarse-grain sediments are poorly sorted and saltation-population dominated. These sedimentary characteristics indicate a dominantly fluvial environment.
2. DU–2 contains massive and/or laminated brown-gray and olive-gray clay and thin yellowish, and brown-gray sandy silt or fine to medium sand. The fine-grain sediments are better sorted than ones of DU–1 and contain one or more suspension populations. *Candona sinuosa*, *Candona compressa*, *Candoniella albicans*, *Candoniella suzini, Darwinula stevensoni* and some freshwater characeae species are identified. In the BH1 and BH2 cores, there are marine-like strata in the upper part of this depositional unit. They consist of thin layers of sandy silt containing mollusk debris; the dominant foraminifera assemblages are *Ammonia multicella*, *Ammonia tepida*, *Elphidium magellanicum*, and *Pseudorotalia variabilis*. These sedimentary characteristics indicate a dominantly lacustrine environment.
3. DU–1 contains yellowish gray and gray sandy silt with olive-gray, gray-brown and reddish clay layers. The dominant foraminifera assemblages are *Ammonia becarii var.*, *Ammonia confertitesta*, *Ammonia limbatobeccarii*, *Ammonia takanabensis*, *Ammonia tepida*, *Cribrononion incertum*, *Elphidium advenum*, *Elphidium limpidum*, *Elphidium magellanicum*, *Elphidium subcrispum*, *Protelphidium granosum*, *Pseudorotalia gaimardii*, *Quinqueloculina* spp., and *Stomoloculina multangula*. The sedimentary sequence of DU–3 is interpreted as corresponding to three major alternations of transgression and regression, which can be correlated to the Cangzhou, Huanghua, and Xianxian Transgressions^10^. The major sedimentary environments are inter-tidal, littoral and deltaic.

According to the results of previous rock magnetic measurements^15^, magnetite is the predominant carrier of the remanent magnetization in the sediments with a much weaker contribution from hematite. Consequently, the following series of demagnetization experiments are conducted in order to isolate the ChRM (Supplementary Table S1): The samples selected for AFD are demagnetized in peak fields up to 80 mT; those selected for TD are demagnetized at temperatures up to 585 °C or 670 °C; and those selected for hybrid demagnetization (TD+AFD+TD) are first demagnetized at 80 °C and 150 °C, then by AFD up to 80 mT, and finally by TD again from 200 °C up to 585 °C or 700 °C (Supplementary Fig. S4). All of the remanent magnetization measurements are conducted using a three-axis cryogenic magnetometer (2G-760 model, 2G Enterprises, USA) installed in field-free space (<300 nT).

Principal component analyses are performed using the Paleomagnetism Data Analysis software (PGMSC, V4.2) developed by Randolph J. Enkin. The ChRM directions are determined by linear least squares fitting^33^ through the origin using at least four continuous demagnetization steps and with a MAD that of less than 15°. Based on the ChRM directions, a magnetozone is defined by at least four continuous ChRM points.

## Additional Information

**How to cite this article**: Yi, L. *et al*. Plio-Pleistocene evolution of Bohai Basin (East Asia): demise of Bohai Paleolake and transition to marine environment. *Sci. Rep.* **6**, 29403; doi: 10.1038/srep29403 (2016).

## Supplementary Material

Supplementary Information
