# Supplementary material for: Plio-Pleistocene evolution of Bohai Basin (East Asia): demise of Bohai Paleolake and transition to marine environment
Source: Sci Rep. 2016 Jul 7;6:29403. doi: 10.1038/srep29403 (PMC4935998; doi:10.1038/srep29403)
Supplement: Supplementary Information [file srep29403-s1.pdf]

Supplementary information

Plio-Pleistocene evolution of Bohai Basin (East Asia): demise of Bohai Paleolake and transition to marine environment

Liang Yi, Chenglong Deng *, Lizhu Tian, Xingyong Xu, Xingyu Jiang, Xiaoke Qiang, Huafeng Qin, Junyi Ge, Guangquan Chen, Qiao Su, Yanping Chen, Xuefa Shi, Qiang Xie, Hongjun Yu, Rixiang Zhu

*To whom correspondence should be addressed. E-mail: cldeng@mail.iggcas.ac.cn

This file includes:

Supplementary Figures S1 to S7;

Supplementary Tables S1 to S4;

Supplementary sedimentary descriptions, including those for Boreholes BH1, BH2, and HLL02;

References.

Supplementary Figures

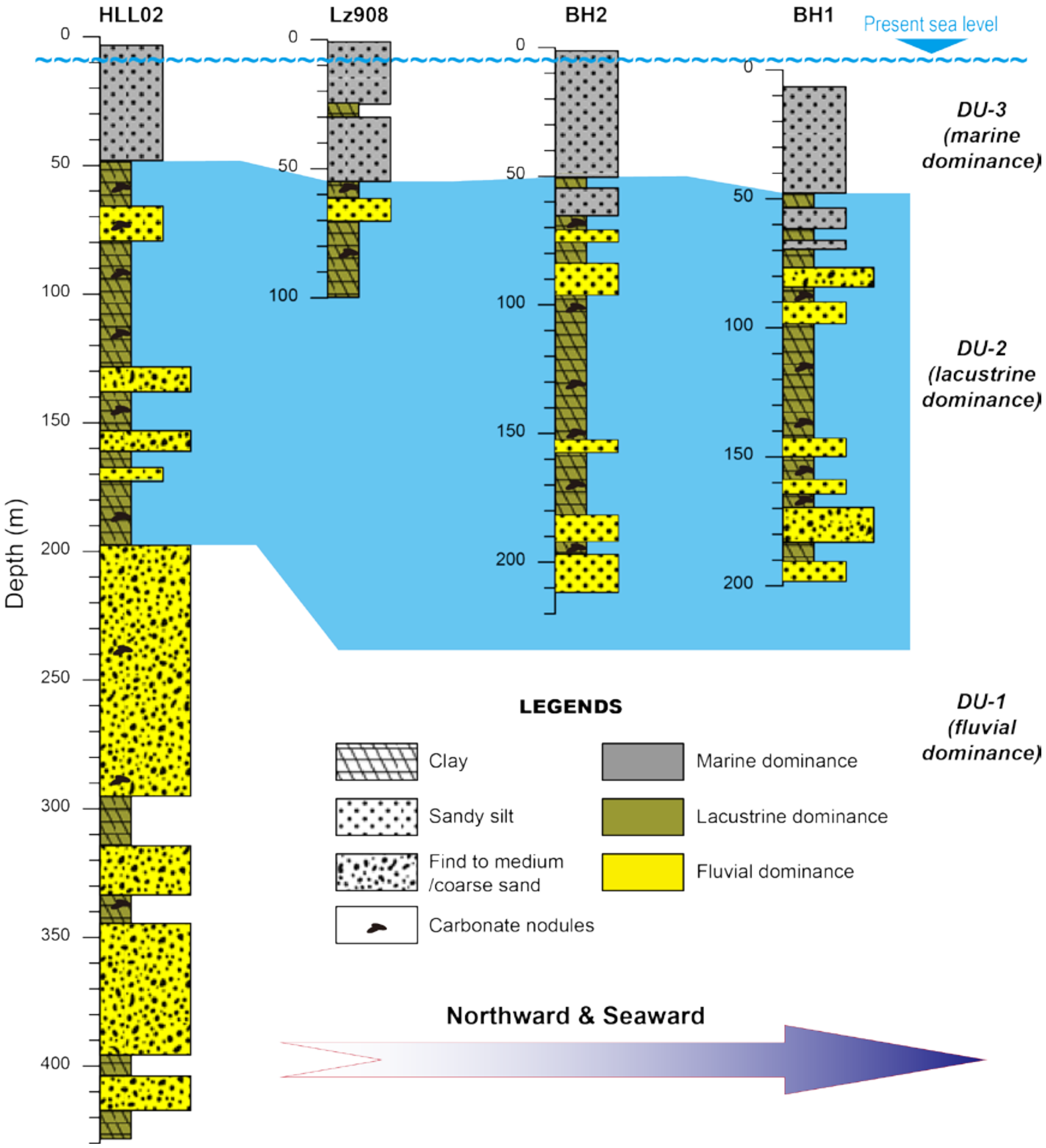

Fig. S1. Lithology of the studied cores.

Borehole BH1 (37°17′N, 119°06′E, and water depth -4 m) was drilled in the winter of 2008. The length of the core is 198.8 m and the recovery rate is 90%. Borehole BH2 (37°10′N, 119°04′E, elevation 3 m a.s.l.) was drilled in the spring of 2009. The drilling position was covered by seawater until the middle of the last century. The length of the core is 228.2 m and the recovery rate is 85%. These two cores were both drilled by the First Institute of Oceanography, State Oceanic Administration of China. The upper coastal and marine sediments of these two cores have been studied using luminescence and radiocarbon dating (Yi et al., 2013). Because Yi et al. (2013) mainly focused on dating the first (the

Holocene) and the second (130–30 ka) transgressions, this study was conducted by revisiting the third transgression and dating the deposition beneath the upper two transgressions using paleomagnetic methods.

Borehole HLL02 was located onshore near the southern coast of the Bohai Sea (37°02′N, 119°08′E, elevation 3 m a.s.l.). The core was drilled to a depth of 425 m below the surface with an average recovery rate of 94% during the summer of 2012 by the Tianjin Center, China Geological Survey, and no report has been previously published on this core. Sedimentary descriptions of all the three cores are listed at the end of this file.

Borehole Lz908 (37°09′N, 118°58′E, elevation 6 m a.s.l.) was drilled in the summer of 2007, with a length of 101.3 m and a recovery rate of 75%. It has been chosen for sedimentary, geochronological and paleoenvironmental studies (Yi et al., 2012a, 2012b, 2015; Yao et al., 2014; Li et al., 2014) and listed here for a reference.

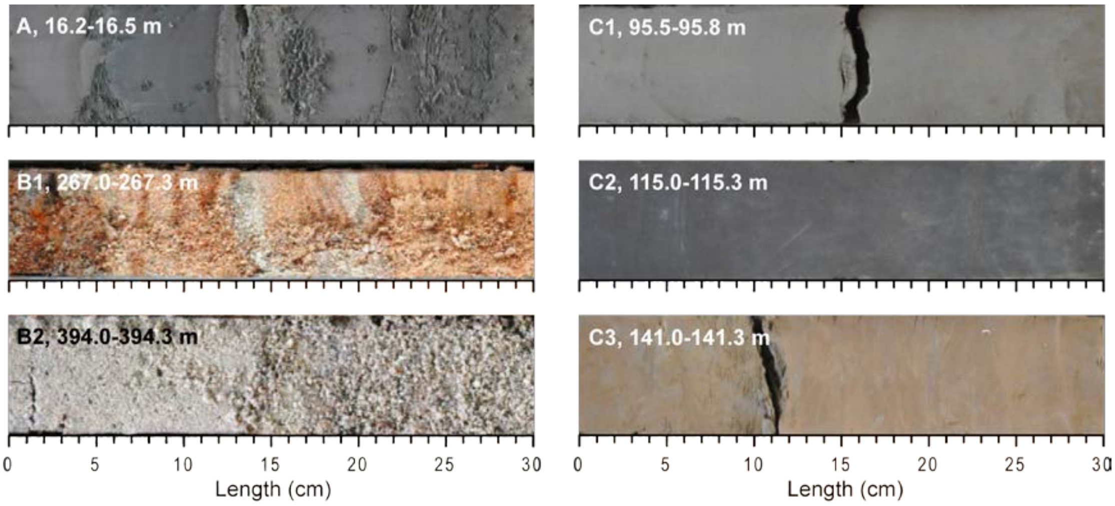

Fig. S2. Sedimentary facies of Borehole HLL02

A: Gray sandy silt with mollusk debris; inferred intertidal facies. B1: Yellowish and snuff-color fine to coarse sand with gravel; inferred alluvial facies. B2: Gray and yellow-gray fine to medium sand; inferred fluvial facies. C1: Olive-gray and gray-brown clay, silty clay; inferred relatively deep lacustrine sub-facies. C2: Gray and dark gray (organic rich) clay; inferred swamp sub-facies. C3, Yellowish and snuff-color clay, silty clay, with carbonate nodules; inferred relatively shallow-water lacustrine sub-facies.

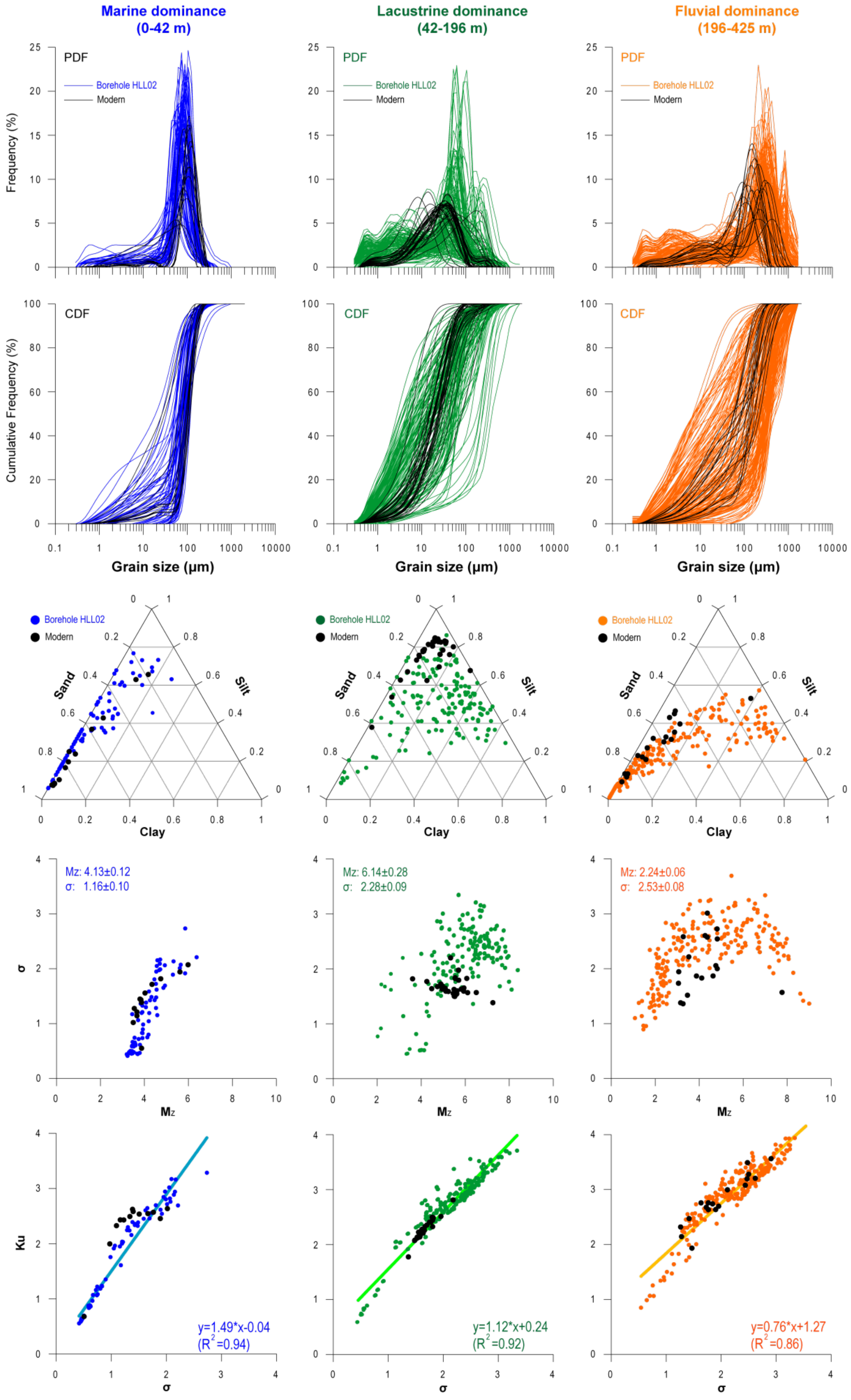

Fig. S3. Sediment grain-size analyses on Borehole HLL02 and modern samples.

A1-C1, probability density function (PDF); A2-C2, cumulative distribution function (CDF); A3-C3, ternary diagrams between clay, silt and sand components; A4-C4, relationship between mean and sorting (Φ values) with their expectations; A5-C5, relationship between sorting and kurtosis (Ku) (Φ values). All the curves and relationships show a significant difference between the marine, lacustrine and fluvial intervals of Borehole HLL02, and a clear similarity between borehole and modern samples.

A total of 403 grain-size samples are collected from Borehole HLL02. The samples are filtered to remove coarse particles (> 2mm), pretreated with 10–20 mL of 30% H_2O_2 to remove organic matter, washed with 10% HCl to remove carbonates, rinsed with deionized water, and then placed in an ultrasonic vibrator for several minutes to facilitate dispersion. Fifty grain-size classes between 0.2 and 2000 μm are determined using a Microtrac S3500 Particle Size Analyzer in the Tianjin Center, China Geological Survey.

Black lines and dots in figure are from modern samples. The marine sediments are from the southwest of Laizhou Bay near to the study area (Yi et al., 2012a). The lacustrine sediments are from the Lugu Lake, Yunnan Province of China provided by Dr. Hao Long from Nanjing Institute of Geography and Limnology, Chinese Academy of Sciences, and from the Nihewan Basin, Hebei Province of China provided by Dr. Junyi Ge. The fluvial sediments are from the Xiaoqinghe River (Fig. 1 in the text) and the Qiantang River, Zhejiang Province of China, collected by the leading author.

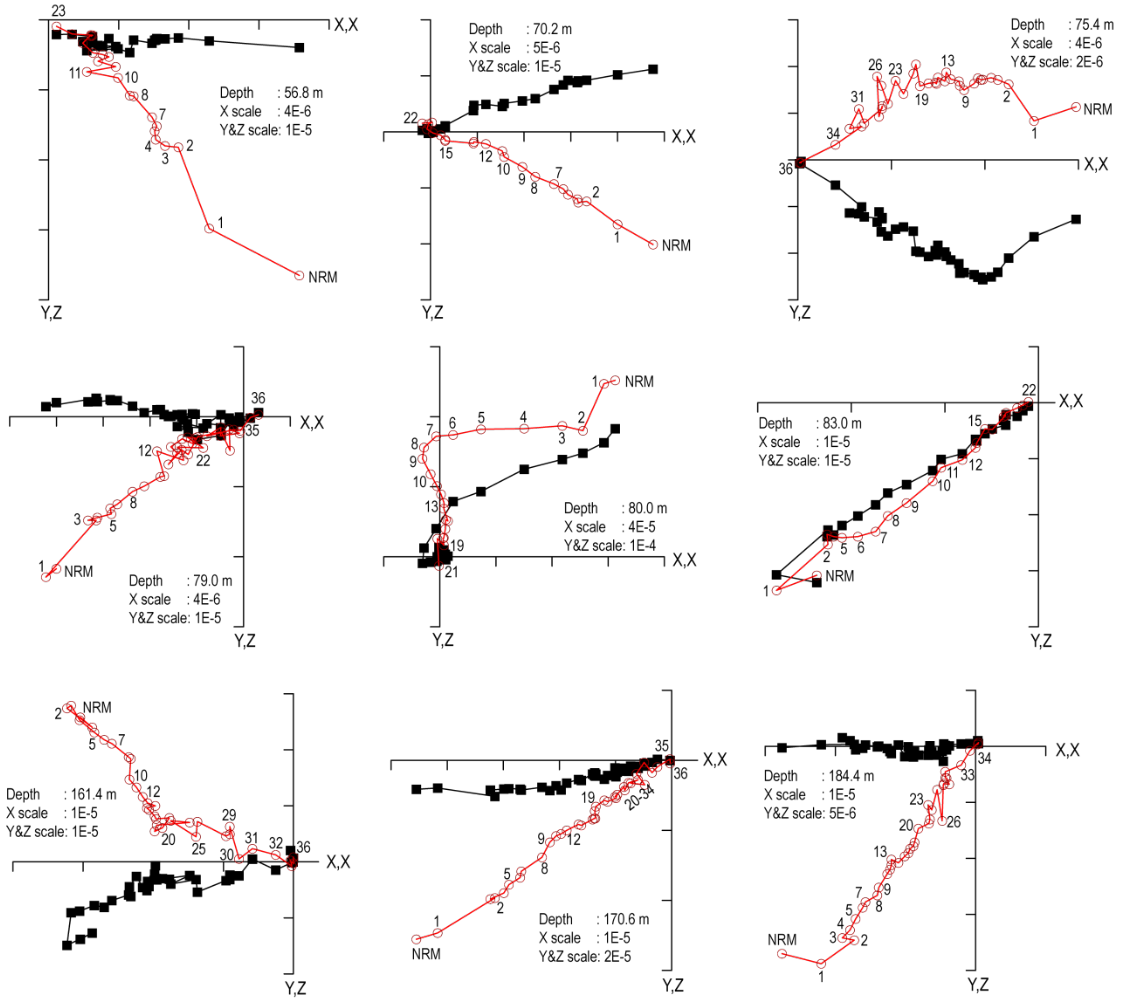

Fig. S4. Orthogonal projections of representative progressive alternating field demagnetization from the BH1 core based on a hybrid demagnetization process (see Table S1).

Notes: The solid (open) circles represent the horizontal (vertical) planes. NRM is the natural remanent magnetization. Numbers indicate demagnetization steps. 1: 80°C; 2: 150°C; 3: 5 mT; 4: 10 mT; 5: 15 mT; 6: 20 mT; 7: 25 mT; 8: 30 mT; 9: 35 mT; 10: 40 mT; 11: 45 mT; 12: 50 mT; 14: 60 mT; 15: 70 mT; 16: 200°C; 17: 250°C; 18: 300°C; 19: 350°C; 20: 400°C; 21: 450°C; 22: 500°C; 23: 525°C; 24: 550°C; 25: 570°C; 26: 585°C; 27: 610°C; 28: 620°C; 29: 630°C; 30: 640°C; 31: 650°C; 32: 660°C; 33: 670°C; 34: 680°C; 35: 690°C; and 36: 700°C.

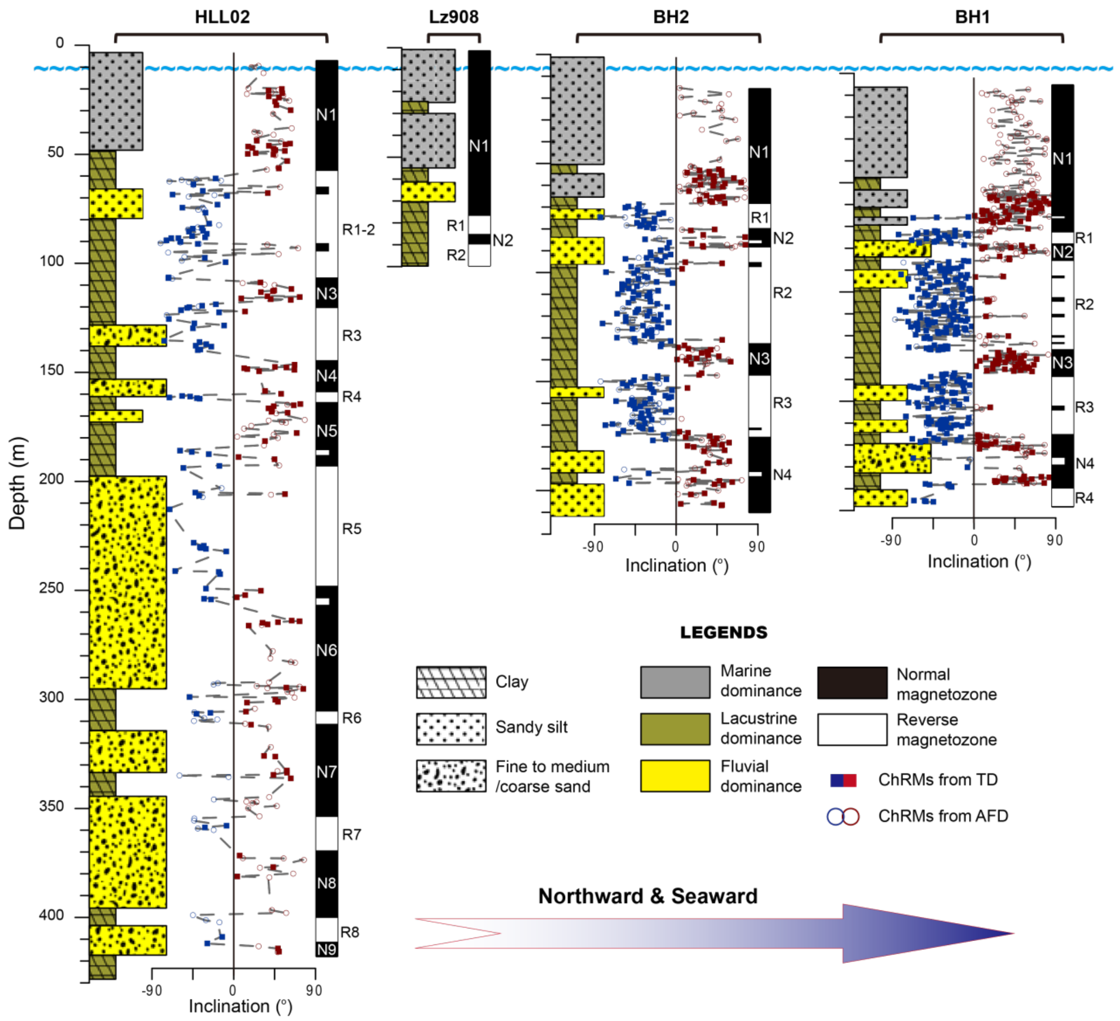

Fig. S5 Magnetostratigraphic results for the studied cores

Borehole BH1. Eight magnetozones are recognized: four with reverse polarity, R1 (71–77 m), R2 (85–126 m), R3 (136–164 m), and R4 (187–196 m); and four with normal polarity, N1 (0–71 m), N2 (77–85 m), N3 (126–136 m), and N4 (164–187 m).

Borehole BH2. Seven magnetozones are recognized: three with reverse polarity, R1 (69–81 m), R2 (89–134 m), and R3 (147–175 m); and four with normal polarity, N1 (0–69 m), N2 (81–89 m), N3 (134–147 m), and N4 (175–207 m).

Borehole HLL02. Seventeen magnetozones are recognized: seven with reverse polarity, R1-2 (57–108 m), R3 (118–146 m), R4 (160–164 m), R5 (192–250 m), R6 (305–311 m), R7 (354–371 m), and R8 (398–412 m); and eight with normal polarity, N1 (0–57 m), N3 (108–118 m), N4 (146–160 m), N5 (164–192 m), N6 (250–305 m), N7 (311–354 m), N8 (371–398 m), and N9 (412–416 m). Note that the magnetic declinations are arbitrary, thus only the magnetic inclination data were used to define magnetic polarity.

Unlike the magnetozones of Borehole BH1 and BH2, there are two reversed polarity intervals (R6 and R7), correlating with the intervening subchrons within the Gauss chron and C3n.2r chron, which comprise relative narrow depth intervals. The lithology of these reversed magnetozones consists of clay and silt, which probably indicates a relatively low sedimentation rate and is compatible with their thinness.

According to the International Geomagnetic Reference Field (IGRF12) model (data covering 1590-2015 AD) (http://www.ngdc.noaa.gov/geomag/), the theoretical value of magnetic inclination at the drilling site (119°E, 37°N) is variable, ranging from 42° to 55°.

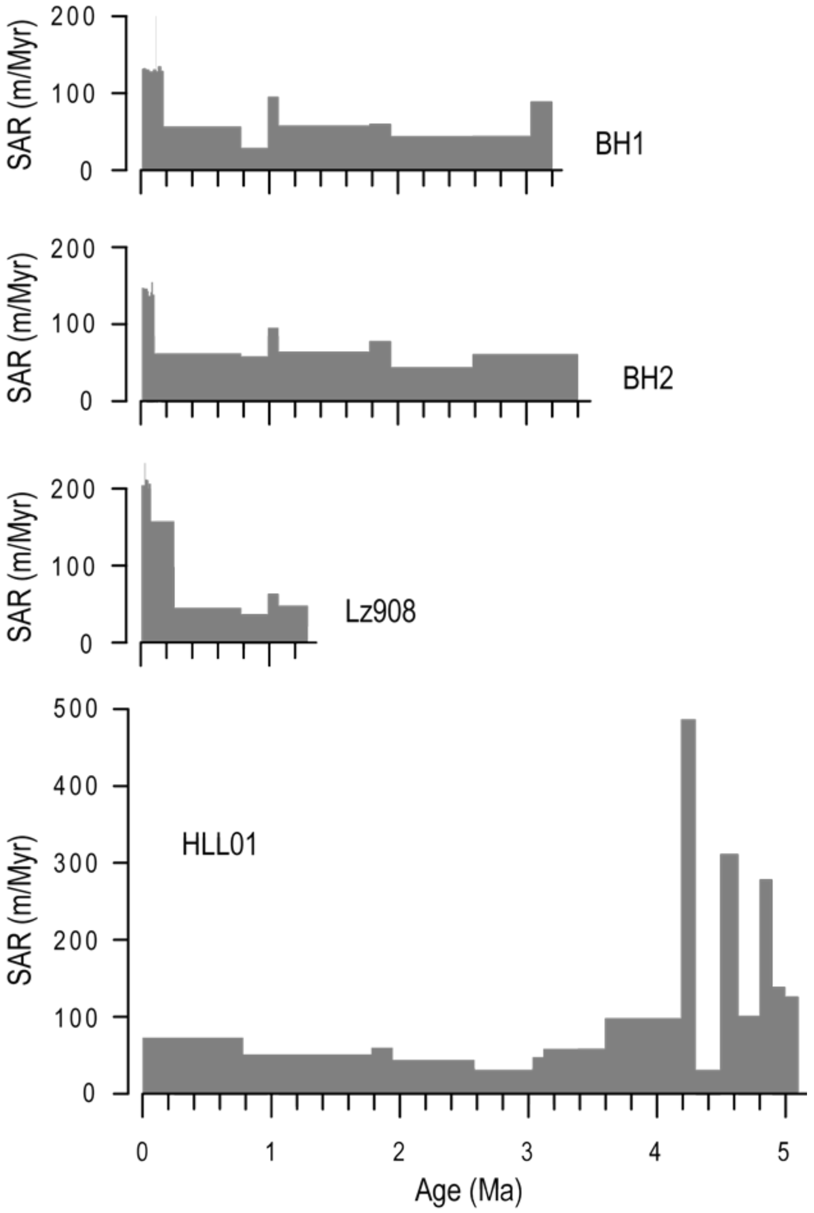

Fig. S6. Variations in sediment accumulation rates (SARs) in the studied cores (see Table S2)

For the lower interval of lacustrine and fluvial dominance, we used the paleomagnetic age constraints based on correlation with ATNTS2012 (Hilgen et al., 2012) to estimate the SAR changes; and for the upper interval of marine influence, luminescence ages were used (Table S4). The SARs exhibit a large range of variation from 30-300 m/Myr, with relatively rapid sedimentation prior to the late Pliocene. The SARs decrease from the late Pliocene to the late Pleistocene and then increase in the late Pleistocene. Since the SARs were relatively uniform within each stage, we used linear regressions to characterize the average SAR values. The data from Lz908 core are from Yi et al. (2015) and are listed here for reference.

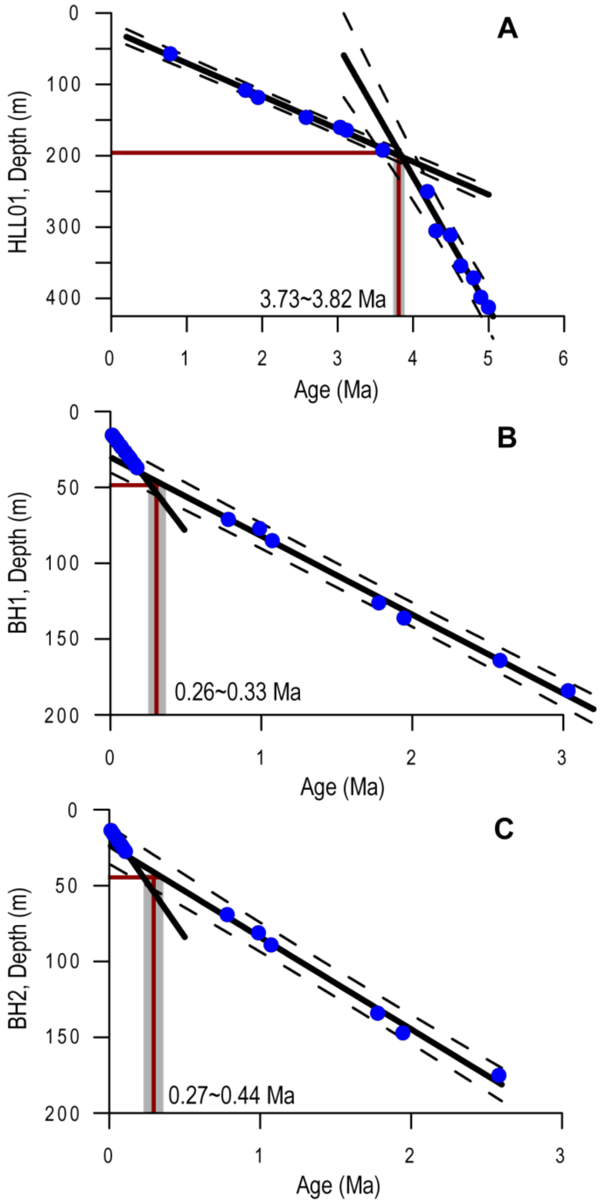

Fig. S7. Linear relationships of age-depth model (A, HLL02 core; B, BH1 core; C, BH2 core)

A, In Borehole HLL02, backward extrapolation of the ages of the lacustrine-dominated phase and the forward extrapolation of the fluvial-dominated phase intersect at 3.73-3.82 Ma; and this is consistent with the age estimates of the sedimentary transition at 196 m. Although there are some uncertainties in the correlation of the magnetozones (Table S3), the consistency between the two extrapolations indicates that they are their reliable.

B and C, the backward extrapolation of the OSL-based age-depth models for Borehole BH1 and BH2 and the forward extrapolation of the paleomagnetic ages intersect at 0.26-0.44 Ma. The OSL data of Yi et al. (2013) are listed in Table S4.

Supplementary Tables

Table S1 Numbers of samples from the three studied cores used for different types of demagnetization

Cores	Methods	Lab.	Total Samples	Number of ChRM	Ratio
**BH1**	AFD (1)	IEE (4)	282	162	57%
	Hybrid (2)	IGG (5)	497	418	84%
		**Total**	**779**	**580**	**74%**
**BH2**	AFD	IEE	254	118	46%
	Hybrid	IGG	204	183	90%
	TD (3)	IGG	48	43	89%
		**Total**	**506**	**344**	**68%**
**HLL02**	AFD	IGG	206	116	56%
	TD	IGG	265	159	60%
		**Total**	**471**	**275**	**58%**

Notes: (1) AFD, alternating field demagnetization; Steps: NRM, 5, 10, 15, 20, 25, 30, 35, 40, 45, 50, 60, 70, and 80 mT. (2) Hybrid steps: NRM, 80°C, 150°C, 5 mT, 10 mT, 15 mT, 20 mT, 25 mT, 30 mT, 35 mT, 40 mT, 45 mT, 50 mT, 60 mT, 70 mT, 200°C, 250°C, 300°C, 350°C, 400°C, 450°C, 500°C, 525°C, 550°C, 570°C, 585°C, 610°C, 620°C, 630°C, 640°C, 650°C, 660°C, 670°C, 680°C, 690°C, and 700°C. (3) TD, thermal demagnetization; Steps: NRM, 80, 150, 200, 250, 300, 350, 400, 450, 500, 520, 540, 560, 585, 600, 620, 640, and 670°C. (4) Paleomagnetism Laboratory, Institute of Earth Environment, Chinese Academy of Sciences. (5) Paleomagnetism and Geochronology Laboratory, Institute of Geology and Geophysics, Chinese Academy of Sciences.

Table S2 Results of One-Way ANOVA of the inclination difference produced by AFD and TD methods

Components		Variance	Sum of Squares	Mean Square	F value	Sig. level (1)	Data Distribution (2)
BH1	Positive inclination	Between Groups	1538	1538	3.27	0.07	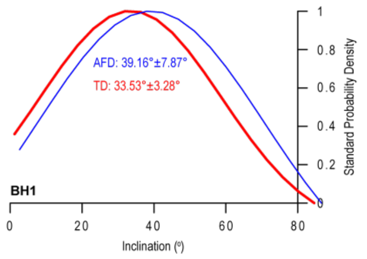
		Within Groups	141542	470			
		Total	143080				
	Negative inclination	Between Groups	3	3	0.01	0.93	
		Within Groups	111537	374			
		Total	111540				
BH2	Positive inclination	Between Groups	<0.1	<0.1	<0.01	0.99	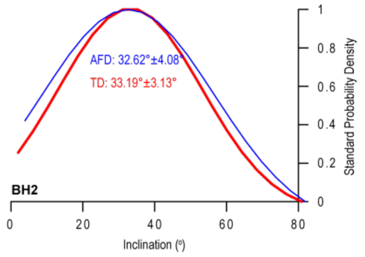
		Within Groups	58243	357			
		Total	58243				
	Negative inclination	Between Groups	171	171	0.55	0.46	
		Within Groups	59969	312			
		Total	60141				
HLL02	Positive inclination	Between Groups	20	20	0.06	0.81	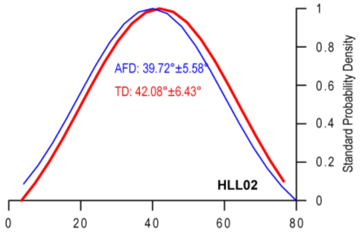
		Within Groups	39765	340			
		Total	39785				
	Negative inclination	Between Groups	306	102	0.31	0.82	
		Within Groups	90639	334			
		Total	90945				

Notes: (1), All differences displayed in the table are insignificant at the *p*<0.05 level. (2), A Gaussian function was fitted to the distribution of the absolute values of the inclination data.

Table S3 Chronostratigraphical framework for the southern Bohai Sea

Phase [1]	Chrons/description		Age [2] (Ma)	HLL02 [5] Depth (m)	Lz908 [6] Depth (m)	BH2 Depth (m)	BH1 Depth (m)
III	Holocene Transgression		0.010 [3]	8	10	10	10
	Termination of the BHPL		0.260 [4]	42	54	50	47
II	C1n	Bottom	0.781	57	77.5	69	71
	C1r.1n	Top	0.988		85.1	81	77
		Bottom	1.072		90.4	89	85
	C2n	Top	1.778	108		134	126
		Bottom	1.945	118		147	136
	C2An.1n	Top	2.581	146		175	164
		Bottom	3.032	160			184
	C2An.2n	Top	3.116	164			
		Bottom	3.207				
	C2An.3n	Top	3.330				
		Bottom	3.596	192			
I	C3n.1n	Top	4.187	250			
		Bottom	4.300	305			
	C3n.2n	Top	4.493	311			
		Bottom	4.631	354			
	C3n.3n	Top	4.799	371			
		Bottom	4.896	398			
	C3n.4n	Top	4.997	412			

Notes: (1) Phases I, II and III are labeled in Fig. 3. (2) The age dataset is from Hilgen et al. (2012). (3) The Holocene transgression has been extensively studied during the past few decades and no major debates are reported. (4) The age of termination of the BHPL was determined by Yi et al. (2015) and further tested in Supplementary Fig. S7. (5) Because there is no other evidence for cross-checking the correlation between the magnetozones and the ATNTS2012 chrons during the late Miocene and early Pliocene, uncertainties remain in this correlation. An evaluation of their reliability is illustrated in Supplementary Fig. S7 (see caption for explanation). (6) Data for Borehole Lz908 are from Yi et al. (2015).

Table S4 OSL ages for Boreholes BH1 and BH2 [1]

Sample ID	Depth (m)	Age (ka)	Interval (ka) [2]
BH1-8A	16.3	9.65±0.81	7-12
BH1-9A	18.7	60±5	45-75
BH1-10A	19.5	47±4	38-56
BH1-11A	22.5	92±9	65-119
BH1-11B	23.4	70±6	52-88
BH1-13B	26.6	109±12	73-145
BH1-14B	29.3	123±15	78-168
BH1-15A	29.5	106±10	83-125
BH1-15B	31.3	111±11	78-144
BH1-17B	34.6	133±17	82-184
BH1-19A	36.9	144±14	102-186
BH2-9B	13.5	8.87±0.73	7-11
H2-10B	16.0	19±2	10-28
BH2-12A	19.8	55±5	46-64
BH2-13	20.8	74±6	56-92
BH2-14B	22.8	75±7	54-96
BH2-15	23.8	80±9	53-107
BH2-16	25.5	125±11	92-158
BH2-17B	27.3	128±12	92-164

Notes: (1) OSL ages are published in Yi et al. (2013). (2) A 99.7% confidence interval is used for the OSL ages.

Supplementary sedimentary descriptions

Borehole BH1

1. 6.3-25.7 m, yellow-brown, yellowish and gray sandy silt and fine sand with mollusk debris, high water content.
2. 25.7-44.4 m, two alternations between yellowish and yellow-brown find sand (2-3 m) and sandy silt and gray-brown and dark-gray sandy silt (3-5 m), with mollusk debris in the fine-sand layers.
3. 44.4-47.2 m, yellowish and yellow-brown sandy silt and fine sand with mollusk debris, high water content.
4. 47.2-54.8 m, snuff-color, olive-gray, gray-brown, mahogany-color clay with small carbonate nodules, compact.
5. 54.8-61.6 m, four alternations of yellowish and yellow-brown sandy silt and find sand in the upper part (3-5 m), yellow-brown and snuff-color clay (1-2 m) in the middle part, and olive-gray, gray and dark gray clay (2-3 m) in the lower part. Laminations occur within the olive-gray clay and the yellowish sandy silt, and small carbonate nodules in the snuff-color clay.
6. 79.9-84.6 m, yellowish and yellow-brown sandy silt and find sand with abundant mollusk debris.
7. 84.6-86.3 m, dark-gray and gray clay.
8. 86.3-86.8 m, snuff-color and yellowish fine to medium sand.
9. 86.8-95.6 m, three alternations between olive-gray and gray clay (~1 m) and snuff-color and yellow-brown clay with carbonate nodules (~2 m), very compact.
10. 95.6-97.6 m, yellowish sandy silt and fine sand, high water content.
11. 97.6-98.7 m, gray-brown and dark-gray clay, very compact, with occasional carbonate nodules.
12. 98.7-100.9 m, laminated gray and dark-gray clay (10-30 cm) with yellowish sandy silt and fine sand (~10 cm).
13. 100.9-102.7 m, yellowish sandy silt and fine sand.
14. 102.7-104.6 m, snuff-color and yellow-brown clay with small carbonate nodules, with gray-brown sandy silt and fine sand in the lower part.
15. 104.6-110.6 m, three alternations of olive-gray and gray clay and snuff-color and yellow-brown clay, with occasional carbonate nodules, very compact.
16. 110.6-119.4 m, two alternations of dark-gray and gray-brown clay with olive gray and gray clay, with some carbonate nodules, very compact.
17. 119.4-123.2 m, laminated gray and dark-gray clay (10-30 cm) with yellowish sandy silt (~10 cm).
18. 123.2-135.2 m, four alternations between snuff-color and yellow-brown clay with carbonate nodules and olive-gray and gray clay, very compact.
19. 135.2-139.3 m, yellowish sandy silt and fine sand.
20. 139.3-145.9 m, two alternations of thick olive-gray and gray clay and thin snuff-color and yellow-brown clay with occasional carbonate nodules, very compact.
21. 145.9-147.2 m, yellowish sandy silt with occasional micro-lamination.

22. 147.2-147.8 m, gray-brown and gray clay with occasional gravel clasts.
23. 147.8-152.3 m, two alternations of olive-gray and gray clay and snuff-color and yellowish clay with carbonate debris, very compact.
24. 152.3-170.1 m, two alternations of yellowish and yellow-brown sandy silt in the upper part, gray gray-brown clay in the middle, and snuff-color and yellowish clay with carbonate nodules in the lower part.
25. 170.1-198.8 m, two alternations of thick yellowish fine to medium sand in the upper part (6-10 m) and thin snuff-color and yellowish clay with small carbonate nodules in the lower part (3-5 m).

Borehole BH2

1. 0.3-12.0 m, yellow-brown, yellowish and gray sandy silt and fine sand with mollusk debris, high water content.
2. 12.0-13.8 m, dark-gray clay with plant and mollusk debris.
3. 13.8-21.3 m, yellowish and yellow-brown sandy silt and fine sand with mollusk debris, high water content.
4. 21.3-24.6 m, yellowish and yellow-brown clay with small carbonate nodules.
5. 24.6-34.5 m, yellow-brown, yellowish and gray sandy silt and fine sand with mollusk debris, high water content.
6. 34.5-35.8 m, dark-gray clay with mollusk debris.
7. 35.8-50.4 m, yellowish and yellow-brown sandy silt and fine sand with mollusk debris, high water content.
8. 50.4-52.9 m, dark-gray and gray-brown clay, compact.
9. 52.9-65.9, yellow-brown, yellowish and gray sandy silt and fine sand with occasional mollusk debris.
10. 55.9-80.1 m, two alternations of olive-gray, gray-brown and gray clay and snuff-color and yellowish clay with occasional carbonate nodules.
11. 80.1-91.8 m, three alternations of yellowish and yellow-brown sandy silt and fine sand with thin snuff-color and yellow-brown clay with carbonate nodules, and olive gray clay, very compact.
12. 91.8-132.3 m, seven alternations of olive-gray and gray clay (1-3 m) and snuff-color and yellow-brown clay (1-5 m), very compact. Carbonate nodules (diameter, 1-5 cm) distributed throughout the section.
13. 132.3-153.6 m, two alternations of gray and dark-gray clay (2-5 m) in the upper part, olive-gray and gray-brown clay (1-3 m) in the middle, and snuff-color and yellowish clay with carbonate nodules in the lower part (~2 m).
14. 153.6-156.3 m, gray-brown and yellow-brown sandy silt and fine sand.
15. 156.3-182.7 m, two alternations between snuff-color and yellow-brown clay with carbonate nodules and olive-gray and gray-brown clay, very compact.
16. 182.7-194.6 m, two alternations of thick yellowish and yellow-brown fine to medium sand and

thin snuff-color and yellow-brown clay with carbonate nodules.

17. 194.6-198.2 m, olive-gray and gray clay with small carbonate nodules.
18. 198.2-208.4 m, yellowish and yellow-brown fine to medium sand.
19. 208.4-212.1 m, laminated yellowish and yellow-brown fine to medium sand with snuff-color and yellow-brown clay.
20. 212.1-228.2 m, thick yellowish and snuff-color fine to coarse sand.

Borehole HLL02

1. 0-11.5 m, yellowish, snuff-color and yellowish gray sandy silt and fine sand, with mollusk debris, high water content.
2. 11.5-35.0 m, laminated yellowish and snuff-color sandy silt and clay, with mollusk debris and small carbonate nodules, high water content, in the upper part; massive yellowish sandy silt and fine sand in the lower part, and 0.5-m-thick snuff-color and yellowish clay with small carbonate nodules at the bottom.
3. 35.0-42.1 m, yellowish and snuff-color sandy silt and fine sand with mollusk debris, and with thin organic rich sediment at the bottom.
4. 42.1-50.0 m, thick yellow-brown and snuff-color clay with small carbonate nodules.
5. 50.0-57.0 m, yellowish and snuff-color sandy silt and fine sand with a high water content, and gray and gray-brown clay with laminated organic-rich clay at the bottom.
6. 57.0-61.0 m, thick yellowish and snuff-color clay with carbonate nodules.
7. 61.0-78.5 m, two alternations of olive-gray and gray-brown clay and yellowish and snuff-color silty clay with carbonate nodules.
8. 78.5-79.6 m, yellowish and snuff-color fine to medium sand, moderately sorted.
9. 79.6-82.7 m, olive-gray and gray clay with carbonate nodules.
10. 82.7-89.3 m, yellowish and orange color sandy silt and fine sand with mollusk debris.
11. 89.3-105.8 m, three alternations between yellowish and snuff-color clay with carbonate nodules in the upper part, and yellowish and orange color fine to medium sand with occasional gravel clasts in the lower part.
12. 105.8-130.4 m, three alternations of thick yellowish and snuff-color clay with carbonate nodules and olive-gray and gray-brown clay.
13. 130.4-180.0 m, four alternations between yellowish, snuff-color and orange color fine to medium sand with carbonate nodules and occasional gravel clasts (diameter < 3cm) in the upper part, and yellowish, snuff-color and yellow-gray silty clay with small carbonate nodules, very compact.
14. 180.0-184.8 m, laminated yellowish and orange color fine to medium sand with olive-gray and gray clay.
15. 184.8-195.9 m, thick gray and light gray clay with occasional carbonate nodules.
16. 195.9-202.7 m, thick yellowish and orange color fine to coarse sand and gravel clasts.
17. 202.7-207 m, thick gray and light gray clay with occasional carbonate nodules.

18. 207-226.3 m, laminated yellowish and snuff-color fine to medium sand.
19. 226.3-232.9 m, laminated yellowish clay with snuff-color and yellow-brown clay, with carbonate nodules and fine gravel clasts.
20. 232.9-247.7 m, two alternations between yellowish and orange-color fine to medium sand with thin clay layers and occasional gravel clasts in the upper part, and gray, light gray and yellowish gray silty clay with small carbonate nodules in the lower part.
21. 266.6-295.3 m, yellowish and orange color fine to medium sand with thin silty clay.
22. 295.3-296.9 m, yellowish and snuff-color silty clay with charcoal and carbonate nodules.
23. 296.9-304.1 m, yellowish and snuff-color sandy silt and fine sand.
24. 304.1-309.0 m, light brown and light gray silty clay.
25. 309.0-331.0 m, laminated yellowish and orange color fine to medium sand with light gray and yellowish silty clay, and with coarse sand and gravel clasts at the bottom.
26. 331.0-337.6 m, light gray and gray orange color fine to medium sand with occasional gravel clasts.
27. 337.6-376.0 m, laminated gray and light gray fine to medium sand with yellowish and orange-color silty clay.
28. 376.0-380.4 m, laminated light gray silty clay with yellowish fine sand, with fine gravel clasts.
29. 380.4-395.8 m, thick gray and light gray fine to coarse sand with occasional gravel clasts, poorly sorted.
30. 395.8-399.5 m, light gray clay with fine gravel clasts.
31. 399.5-408.3 m, thick light yellowish fine to medium sand.
32. 408.3-417.5 m, thick gray, light gray and light yellowish sandy silt and fine sand.
33. 417.5-425.0 m, light gray and gray-white medium to coarse sand, with thin coarse silt to fine sand layers, poorly sorted.

References

Hilgen, F.J., Lourens, L.J., Van Dam, J.A., 2012. The Neogene Period, in: Gradstein, F.M., Ogg, J.G., Schmitz, M.D., Ogg, G.M. (Eds.), The Geological Time Scale 2012. Elsevier BV, pp. 923-978.

Li, Y., Yu, H.J., Yi, L., Su, Q., Hu, K., Xu, X.Y., Wang, J., 2014. Grain-size characteristics and its sedimentary significance of coastal sediments of the borehole Lz908 in the south Bohai Sea (the Laizhou Bay), China. Marine Sciences 38, 107-113.

Yao, J., Yu, H.J., Xu, X.Y., Yi, L., Chen, G., Su, Q., 2014. Paleoenvironmental changes during the late Quaternary as inferred from foraminifera assemblages in the Laizhou Bay. Acta Oceanologica Sinica 33, 10-18.

Yi, L., Deng, C., Xu, X., Yu, H., Qiang, X., Jiang, X., Chen, Y., Su, Q., Chen, G., Li, P., Ge, J., Li, Y., 2015. Paleo-megalake termination in the Quaternary: Paleomagnetic and water-level evidence from south Bohai Sea, China. Sedimentary Geology 319, 1-12.

Yi, L., Lai, Z., Yu, H., Xu, X., Su, Q., Yao, J., Wang, X., Shi, X., 2013. Chronologies of sedimentary changes in the south Bohai Sea, China: constraints from luminescence and radiocarbon dating. Boreas 42, 267-284.

Yi, L., Yu, H., Ortiz, J.D., Xu, X., Chen, S., Ge, J., Hao, Q., Yao, J., Shi, X., Peng, S., 2012a. Late Quaternary linkage of sedimentary records to three astronomical rhythms and the Asian monsoon, inferred from a coastal borehole in the south Bohai Sea, China. Palaeogeography, Palaeoclimatology, Palaeoecology 329-330, 101-117.

Yi, L., Yu, H.J., Ortiz, J.D., Xu, X.Y., Qiang, X.K., Huang, H.J., Shi, X., Deng, C.L., 2012b. A reconstruction of late Pleistocene relative sea level in the south Bohai Sea, China, based on sediment grain-size analysis. Sedimentary Geology 281, 88-100.
